# Comprehensive Transcriptional Profiling of the Gastrointestinal Tract of Ruminants from Birth to Adulthood Reveals Strong Developmental Stage Specific Gene Expression

**DOI:** 10.1534/g3.118.200810

**Published:** 2018-12-10

**Authors:** Stephen J. Bush, Mary E. B. McCulloch, Charity Muriuki, Mazdak Salavati, Gemma M. Davis, Iseabail L. Farquhar, Zofia M. Lisowski, Alan L. Archibald, David A. Hume, Emily L. Clark

**Affiliations:** The Roslin Institute and Royal (Dick) School of Veterinary Studies, University of Edinburgh, Edinburgh, UK

**Keywords:** gene expression, transcription, sheep, goat, ruminant, gastrointestinal tract, development, macrophage, immunity, RNA-Seq

## Abstract

One of the most significant physiological challenges to neonatal and juvenile ruminants is the development and establishment of the rumen. Using a subset of RNA-Seq data from our high-resolution atlas of gene expression in sheep (*Ovis aries*) we have provided the first comprehensive characterization of transcription of the entire gastrointestinal (GI) tract during the transition from pre-ruminant to ruminant. The dataset comprises 164 tissue samples from sheep at four different time points (birth, one week, 8 weeks and adult). Using network cluster analysis we illustrate how the complexity of the GI tract is reflected in tissue- and developmental stage-specific differences in gene expression. The most significant transcriptional differences between neonatal and adult sheep were observed in the rumen complex. Comparative analysis of gene expression in three GI tract tissues from age-matched sheep and goats revealed species-specific differences in genes involved in immunity and metabolism. This study improves our understanding of the transcriptomic mechanisms involved in the transition from pre-ruminant to ruminant by identifying key genes involved in immunity, microbe recognition and metabolism. The results form a basis for future studies linking gene expression with microbial colonization of the developing GI tract and provide a foundation to improve ruminant efficiency and productivity through identifying potential targets for novel therapeutics and gene editing.

Sheep are an important source of meat, milk and fiber for the global livestock sector and belong to one of the most successful groups of herbivorous mammals, the ruminants. Adult sheep have four specialized chambers comprising their stomach: fermentative fore-stomachs encompassing the rumen, reticulum and omasum and the “true stomach”, the abomasum (Dyce *et al.* 2010). The events surrounding the development of the rumen are among the most significant physiological challenges to young ruminants (Baldwin *et al.* 2004). As lambs transition from a milk diet to grass and dry pellet feed the gastrointestinal (GI) tract undergoes several major developmental changes. In neonatal lambs, feeding solely on milk, the fermentative fore-stomachs are not functional and the immature metabolic and digestive systems function similarly to that of a young monogastric mammal, with proteolytic digestion taking place inside the abomasum (Meale *et al.* 2017). At this stage the rumen has a smooth, stratified squamous epithelium with no prominent papillae (Baldwin *et al.* 2004). Suckling causes a reflex action that brings the walls of the reticulum together to form an ‘esophageal’ or ‘reticular’ groove transferring milk and colostrum directly to the abomasum, where it is digested efficiently (Figure 1) (Dyce *et al.* 2010). In neonatal ruminants this is essential to ensure protective anti-bodies in the colostrum are transported intact to the abomasum.

**Figure 1:**
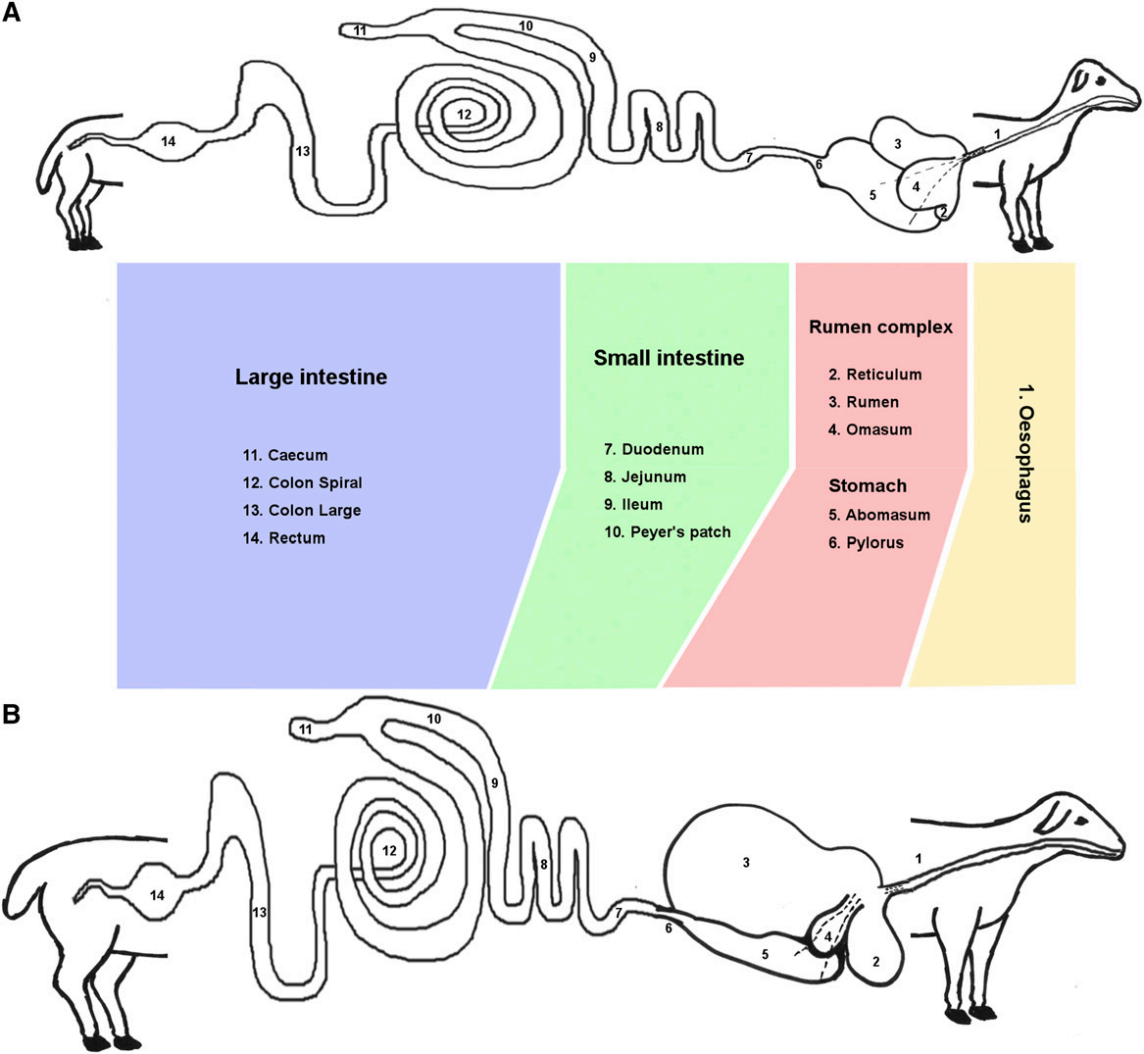
Diagrammatic representation of the morphological changes that occur in the gastrointestinal tract of a sheep during the transition from (A) pre-ruminant to (B) ruminant. The 14 regions sampled for this study are numbered (12 samples from each of the lambs and 2 from the adult animals only – esophagus and rectum). The esophageal groove is indicated with dotted lines.

The introduction of grass and dry feed into the diet (which usually occurs in very small amounts from one week of age) inoculates the rumen with microbes. Microbes proliferate, facilitating the digestion of complex carbohydrates which the adult ruminant relies upon to meet its metabolic needs (Bryant *et al.* 1958). The production of short-chain fatty acids from the digestion of complex carbohydrates by microbes stimulates growth and development of the rumen and reticulum (Bryant *et al.* 1958). The transition from pre-ruminant to ruminant occurs gradually from around 4 weeks of age. The rumen and reticulum are usually fully functional by the time the lamb reaches 8 weeks of age and has a completely grass and dry feed-based diet (Figure 1). The transition results in metabolic changes, as tissues shift from reliance on glucose supplied from milk to the metabolism of short-chain fatty acids as primary energy substrates. While the most dramatic physical changes occurring during development are associated with the rumen epithelium, changes in intestinal mass, immunity and metabolism also occur in response to dietary changes (Baldwin *et al.* 2004). These processes are likely to be intrinsically linked as the GI tract protects the host from toxic or pathogenic luminal contents, while at the same time supporting the absorption and metabolism of nutrients for growth and development (reviewed in (Steele *et al.* 2016; Meale *et al.* 2017)).

Many studies have used quantitative PCR to measure the expression of sets of candidate genes in ruminant GI tract tissues (reviewed in (Connor *et al.* 2010)). RNA-Sequencing (RNA-Seq) technology now provides a snapshot of the transcriptome in real-time to generate global gene expression profiles. This allows us to measure the expression of all protein coding genes throughout the development of the GI tract and associate these expression patterns with immunity, metabolism and other cellular processes at the gene/transcript level. Previous studies have used RNA-Seq to examine links between feed intake and microbial diversity and the development of the rumen (Connor *et al.* 2013; Wang *et al.* 2016; Xiang *et al.* 2016a). Another recent study characterized transcription in the adult rumen complex and GI tract of sheep, linking immune, epithelial and metabolic transcriptomic signatures (Xiang *et al.* 2016b). Similarly, transcriptional analysis of colon, cecum and duodenum from two breeds of sheep highlighted key genes involved in lipid metabolism (Chao *et al.* 2017).

To characterize tissue specific transcription in the GI tract during early development we utilized a subset of RNA-Seq data, from Texel x Scottish Blackface (TxBF) lambs at birth, one week and 8 weeks of age and TxBF adult sheep, from our high resolution atlas of gene expression in sheep (Clark *et al.* 2017b). We characterize in detail the transcriptional signatures in GI tract tissues at each developmental stage and link these to other key biological processes occurring as the lamb develops. We also perform comparative analysis of transcription in the rumen, ileum and colon of one-week old sheep with age-matched goats. A clearer understanding of the transcriptomic complexity that occurs during the transition between pre-ruminant and ruminant will provide a foundation to improve ruminant efficiency and productivity by identifying key genes involved in this process that can be used as targets for gene editing or as novel therapeutics.

## Materials and Methods

### Animals

Approval was obtained from The Roslin Institute and the University of Edinburgh Protocols and Ethics Committees. All animal work was carried out under the regulations of the Animals (Scientific Procedures) Act 1986. Full details of all the sheep used in this study are provided in (Clark *et al.* 2017b) and summarized in Table 1. GI tract tissues were collected from three male and three female adult Texel x Scottish Blackface (TxBF) sheep and nine Texel x Scottish Blackface lambs. Of these nine lambs, three were observed at parturition and euthanised immediately prior to their first feed. Three lambs were euthanised at one week of age pre-rumination (no grass was present in their GI tract) and three at 8 weeks of age once rumination was fully established. All the animals were fed *ad libitum* on a diet of hay and sheep concentrate nuts (16% dry matter), with the exception of the lambs pre-weaning (birth and one week of age) who suckled milk from their mothers. Goat GI tract samples from one-week old pre-weaned male goat kids were obtained from an abattoir.

**Table 1:** Details of animals and samples from the GI tract and other tissues included in this study

Species	Breed	Developmental Stage	Sex	GI Tract Tissues	Reference & ENA Study Accession
**Sheep**	Texel x Scottish Blackface	Birth (neonate/ pre-ruminant)	1 male, 2 females	**Stomach**	Clark *et al.* 2017 (PRJEB19199)
				Abomasum, Pylorus	
**Sheep**	Texel x Scottish Blackface	One Week (transition from pre-ruminant to ruminant)	1 male, 2 females	**Large Intestine**	
				Cecum, Colon Spiral, Colon Large	
**Sheep**	Texel x Scottish Blackface	8 Weeks (ruminant)	2 males, 1 female	**Small Intestine**	
				Duodenum, Ileum, Jejunum, Peyer’s Patch	
**Sheep**	Texel x Scottish Blackface	Adult (GI tract) (2 years)	3 males, 3 females	**Rumen Complex**	
				Omasum, Reticulum, Rumen	
**Sheep**	Texel x Scottish Blackface	Adult (2 years)	3 males, 3 females	Esophageal Mucosa, Esophageal Muscle, Rectum, Liver, Alveolar Macrophages	Clark *et al.* 2017 (PRJEB19199)
**Sheep**	Texel	8 – 9 Months	1 female	Abomasum, Colon, Cecum, Rectum, Rumen, Omentum, Peyer’s Patch	Jiang *et al.* 2014 (PRJEB6169)
**Sheep**	Texel	Adult (>2 years)	1 male, 1 female		
**Goat**	Crossbred	One Week	3 male	Ileum, Rumen, Colon Large, Alveolar Macrophages	This Study (PRJEB23196)

### Tissue collection

In total twelve different regions of the sheep GI tract were sampled, as detailed in Table 1 and illustrated in Figure 1. All post mortems were undertaken by the same veterinary anatomist and tissue collection from the GI tract was standardized as much as possible. All GI tract tissue samples were washed twice in room temperature sterile 1x PBS (Mg^2+^ Ca^2+^ free) (P5493; Sigma Aldrich, Missouri, USA) then chopped into small pieces <0.5cm and transferred to RNAlater preservation solution (AM7021; Thermo Fisher Scientific, Waltham, USA). To maintain RNA integrity, all GI tract tissue samples were harvested within 30 min from the time of death. The goat samples (Table 1) were collected using the same methods as described for sheep. Additional samples were included in the analysis from the rectum, alveolar macrophages, liver and thoracic esophagus (muscle and mucosa) of adult TxBF as described in (Clark *et al.* 2017b) and Texel sheep (Jiang *et al.* 2014) (Table 1).

### RNA extraction and library preparation

We used a TRIzol (15596018; Thermo Fisher Scientific) based RNA extraction method which is described in detail in (Clark *et al.* 2017b). RNA quantity was measured using a Qubit RNA BR Assay kit (Q10210; Thermo Fisher Scientific) and RNA integrity estimated on an Agilent 2200 Tapestation System (Agilent Genomics, Santa Clara, USA) using the RNA Screentape (5067-5576; Agilent Genomics) to ensure RNA quality was of RNA integrity number equivalent (RIN^e^) > 7 (Agilent Technologies 2016). RNA was of sufficient quality from 164 of the 180 samples collected from TxBF sheep. Samples which failed QC included: 3 pylorus, 4 duodenum, 1 ileum, 2 Peyer’s patch, 3 cecum and 1 colon spiral from the adult animals; 1 abomasum sample from a one-week old lamb; and 1 Peyer’s patch from an 8-week old lamb. RNA-Seq libraries were prepared for the remaining 164 samples by Edinburgh Genomics (Edinburgh Genomics, Edinburgh, UK) and run on the Illumina HiSeq 2500 (sheep) and Illumina HiSeq 4000 (goats) sequencing platforms (Illumina, San Diego, USA). The GI tract tissues collected from the 9 TxBF lambs were sequenced at a depth of >25 million strand-specific 125bp paired-end reads per sample using the standard Illumina TruSeq mRNA library preparation protocol (poly-A selected) (Ilumina; Part: 15031047 Revision E). The adult sheep GI tract tissues were also sequenced as above with the exception of the ileum, reticulum and liver which were sequenced using the Illumina TruSeq total RNA library preparation protocol (ribo-depleted) (Ilumina; Part: 15031048 Revision E) at a depth of >100 million reads per sample. The ileum and rumen samples from goats were sequenced at a depth of >30 million strand-specific 75bp paired-end reads per sample using the standard Illumina TruSeq mRNA library preparation protocol (poly-A selected) (Ilumina; Part: 15031047 Revision E). The RNA-Seq libraries for the Texel dataset (Jiang *et al.* 2014) were also prepared by Edinburgh Genomics with RNA isolated using the same method as described in (Clark *et al.* 2017b).

### Data quality control and processing

The raw data for sheep, in the form of .fastq files, was previously released by (Clark *et al.* 2017b) and are deposited in the European Nucleotide Archive (ENA) under study accession number PRJEB19199 (http://www.ebi.ac.uk/ena/data/view/PRJEB19199). The goat data are also deposited in the ENA under study accession number PRJEB23196 (http://www.ebi.ac.uk/ena/data/view/PRJEB23196). Both sets of data were submitted to the ENA with experimental metadata prepared according to the FAANG Consortium metadata and data sharing standards (Harrison *et al.* 2018). Details of all the samples for sheep and goat, with associated data and metadata can also be found on the FAANG Data Portal (http://data.faang.org/) (FAANG 2017). The raw read data from the Texel samples incorporated into this dataset and previously published (Jiang *et al.* 2014) are located in the ENA under study accession PRJEB6169 (http://www.ebi.ac.uk/ena/data/view/PRJEB6169). The RNA-Seq data processing methodology and pipelines used for this study are described in detail in (Clark *et al.* 2017b). For each tissue a set of expression estimates, as transcripts per million (TPM), was obtained using the high-speed transcript quantification tool Kallisto v0.43.0 (Bray *et al.* 2016) with the Oar v3.1 reference transcriptome from Ensembl (Zerbino *et al.* 2018) as an index. Expression estimates for the GI tract dataset were then filtered to remove low intensity signals (TPM < 1). To integrate expression estimates from the two different library types we performed a ratio correction of the TPM values as described in (Bush *et al.* 2017).

### Network cluster analysis

Network cluster analysis of the sheep GI tract dataset was performed using the network visualization tool Graphia Professional (Kajeka Ltd, Edinburgh, UK) (Theocharidis *et al.* 2009; Livigni *et al.* 2018). To determine similarities between individual gene expression profiles a Pearson correlation matrix was calculated for both sample-to-sample and gene-to-gene comparisons and filtered to remove relationships where *r* < 0.81 (sample-to-sample) and *r* < 0.85 (gene-to-gene). Network graphs were constructed by connecting nodes (genes or samples) with edges (where the correlation exceeded the threshold value). To interpret each graph a Markov Cluster algorithm (MCL) (van Dongen & Abreu-Goodger 2012) was applied at an inflation value (which determines cluster granularity) of 2.2. Values were selected empirically so that the graph would connect the majority of genes with a minimum of edges, and have a clearly defined internal structure. Visual examination was used to interrogate the local structure of the graph. Similar samples/tissue types and genes with robust co-expression patterns formed clusters of highly interconnected nodes, implying related functions. To determine if genes within a cluster shared a similar biological function GO term enrichment based on gene ontology (Ashburner *et al.* 2000) was performed using the Bioconductor package ‘topGO’ (Alexa & Rahnenfuhrer 2010). For the gene-to-gene network analysis the top 20 largest clusters were assigned a functional class and sub-class based on GO term enrichment, gene function and the ‘guilt-by-association’ principle (Oliver 2000). Additional data from a small subset of tissues from adult TxBF sheep from (Clark *et al.* 2017b) was included in the gene-to-gene network graph. These tissues were included to compare the transcriptional signatures of GI tract tissues to those from a population of macrophages (alveolar macrophages), a metabolic tissue (liver), and GI tract tissues not sampled in the lambs (esophageal tissue, rectum). The Texel dataset from (Jiang *et al.* 2014) was also included in the gene-to-gene network graph to verify that the expression patterns and clustering of samples were consistent across the two datasets. The sample-to-sample network analysis was used to illustrate transcriptional changes in tissues through each developmental stage (birth, one week, 8 weeks and adult) and included only the dataset from the TxBF sheep.

### Principal component analysis of transcriptional signatures in the developing GI tract

All statistical analysis was carried out in R (v >= 3.0.0) (R Core Team 2014) unless stated otherwise. We used Principal Component Analysis (PCA) to determine whether there were any strong age or tissue related transcriptional patterns observed in the GI tract. The PCA for macrophage associated signatures was performed using FactoMineR v1.41 (Lê *et al.* 2008) with a subset of genes (n = 490) (extracted from the alveolar macrophage clusters [clusters 7 and 10] from the gene-to-gene network graph), center scaled for computation of the principal components (PCs). The top 5 and 10 PCs explained 62.6% and 76.9% of variability in the data respectively. In order to compare the exploratory and discriminative power of the PCs, the categorical data (age and tissue of origin) was then overlaid on the PCs coordinate maps and colored by group. The mean of the PC coordinates for each group were considered as the center of the circle colored-by-group with a confidence interval of 0.95 as the ellipse size.

### Developmental stage specific differential expression analysis for sheep

Differential expression analysis was used to compare gene-level expression estimates from the Kallisto output as TPM across GI tract tissues and developmental time points, and between age-matched sheep and goats. The R/Bioconductor package tximport v1.0.3 was used to import and summarize the transcript-level abundance estimates from Kallisto for gene-level differential expression analysis using edgeR v3.14.0 (Robinson *et al.* 2010), as described in (Soneson *et al.* 2015; Love *et al.* 2017). For RNA-Seq experiments with less than 6 replicates per time point edgeR may be considered the optimal differential expression analysis package (Schurch *et al.* 2016). We selected three tissues (abomasum, rumen and ileum) as representative samples from 3 of the major compartments of the GI tract: the stomach, rumen complex and small intestine, respectively. Gene expression patterns in each tissue were compared between birth and one week, and one week and 8 weeks of age. To investigate the function of the differentially expressed genes we performed GO term enrichment (Ashburner *et al.* 2000) using ‘topGO’ (Alexa & Rahnenfuhrer 2010). Only GO terms with 10 or more associated genes were included.

### Data availability

All data analyzed during this study are included in this published article and its additional files. The raw RNA-sequencing data are deposited in the ENA under study accessions PRJEB19199 (sheep) (https://www.ebi.ac.uk/ena/data/view/PRJEB19199) and PRJEB23196 (goat) (https://www.ebi.ac.uk/ena/data/view/PRJEB23196). Sheep data can also be viewed and downloaded via BioGPS (http://biogps.org/dataset/BDS_00015/sheep-atlas/) where the gene expression estimates for each tissue are searchable by gene name (http://biogps.org/sheepatlas). Metadata for all tissue and cell samples are deposited in the EBI BioSamples database under group identifiers SAMEG317052 (sheep) and SAMEG330351 (goat). The gene expression estimates for the GI tract samples averaged across individuals and for each individual are also available via the University of Edinburgh DataShare Portal at https://datashare.is.ed.ac.uk/handle/10283/3113. Supplemental material available at Figshare: https://doi.org/10.25387/g3.6741575.

## Results and Discussion

### Gene-to-gene network cluster analysis of the GI tract dataset

The dataset includes 164 RNA-Seq libraries in total from the TxBF sheep described above. Network cluster analysis of the GI tract data were performed using Graphia Professional (Theocharidis *et al.* 2009; Livigni *et al.* 2018). TPM estimates from Kallisto averaged across biological replicates (where possible, 3 sheep per developmental stage) for the GI tract dataset were used to generate the network cluster graph. The full version of this averaged dataset was published with the sheep gene expression atlas and is available for download through the University of Edinburgh DataShare portal (http://dx.doi.org/10.7488/ds/2112). A version including only the TPM estimates for GI tract tissues, alongside alveolar macrophages, thoracic esophagus (mucosa and muscle) and liver is included here as Table S1.

**Figure 2:**
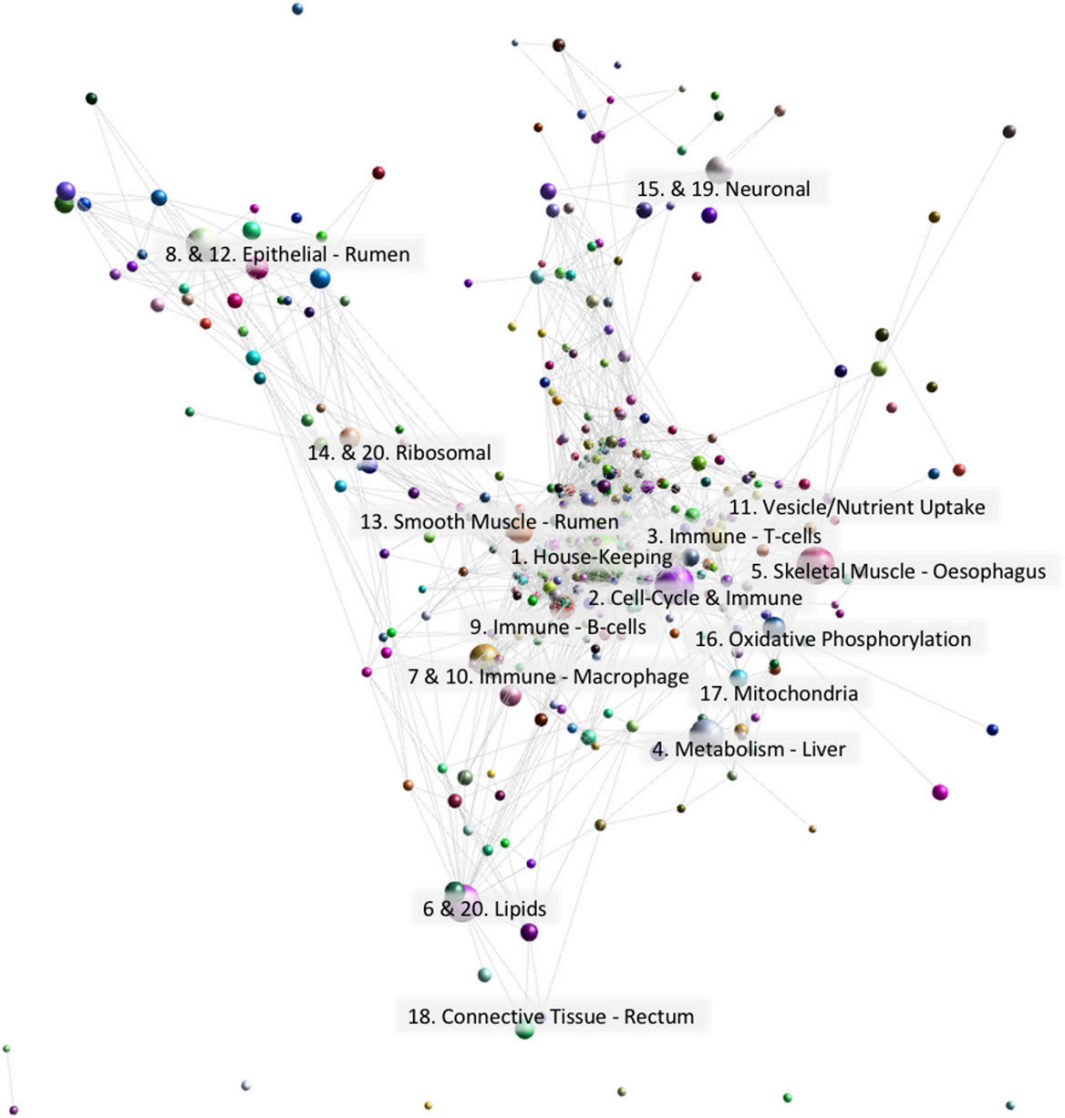
Gene-to-gene network graph of sheep GI tract tissues alongside alveolar macrophages, esophageal tissue and liver with the nodes collapsed by class to illustrate the relative size of each cluster. The top 20 largest clusters are annotated by function. Created using Graphia Professional with parameters Pearson’s R = 0.85, MCLi = 2.2, Minimum Component Size = 2, Minimum Cluster Size = 2.

The dataset was clustered using a Pearson correlation co-efficient threshold of *r* = 0.85 and MCL (Markov Cluster Algorithm (Gough *et al.* 2001)) inflation value of 2.2. The gene-to-gene network graph comprised 13,035 nodes (genes) and 696,618 edges and was highly structured comprising 349 clusters of varying size (Figure 2). Genes found in each cluster are listed in Table S2. Genes in Table S2 labeled ‘assigned to’ were annotated using an automated pipeline described in (Clark *et al.* 2017b). Clusters 1 to 20 (numbered in order of size in Figure 2; cluster 1 being the largest, comprised of 1724 genes) were annotated visually and assigned a broad function. Validation of function was performed using GO term enrichment (Alexa & Rahnenfuhrer 2010) for molecular function, cellular component and biological process (Table S3).

### Complexity of cell types within the GI tract is reflected in gene-to-gene network clustering

The GI tract is a highly complex organ system in ruminants with region-specific cellular composition. This complexity is illustrated by the highly structured gene-to-gene network graph of GI tract tissues (Figure 2). Other studies have characterized in detail the transcriptional signatures in the GI tract of adult sheep (Xiang *et al.* 2016a; Xiang *et al.* 2016b) and as such we will only broadly describe the clusters here and focus instead on developmental stage-specific transcriptional patterns. As is typical of large gene expression datasets from multiple tissues cluster 1, the largest cluster, was comprised largely of ubiquitously expressed ‘house-keeping’ genes, encoding proteins that are functional in all cell types. Enriched GO terms for genes within cluster 1 were for general cellular processes and molecular functions performed by all cells including RNA-processing (*P* = <1x10^−30^) and histone binding (*P* = 3.9x10^−8^) (Table S3).

Although cluster 1 exhibited a general expression pattern the rest of the clusters exhibited tissue/cellular process specific gene expression patterns, with the majority including genes associated with more than one cell type/cellular process. The lamina propria, one of the three layers of the mucosa, which lies beneath the epithelium and lines the majority of the GI tract, is made up of different cell types including endothelial, immune and connective tissues (Takahashi-Iwanaga & Fujita 1985). As a consequence, expression signatures of components of the lamina propria were common across the network graph for sheep. This type of expression pattern in the GI tract was also observed in pig (Freeman *et al.* 2012).

The GI tract of the sheep is lined with epithelium, and the rumen in particular exhibits a strong epithelial signature, which differentiates it transcriptionally from other tissues (Xiang *et al.* 2016a; Xiang *et al.* 2016b). Genes in clusters 8 and 12, expressed in the rumen, exhibited a typical epithelial signature, including genes in the KRT superfamily and enrichment for GO terms such as keratinocyte differentiation. Genes in cluster 5 were expressed in thoracic esophageal skeletal muscle tissue and associated with muscle fiber development (*P* = 1.3x10^−10^) and skeletal muscle tissue development (*P* = 4.4x10^−12^). Several skeletal muscle specific genes were found within this cluster including *ACTA1*, which is associated with skeletal muscle function and encodes a product belonging to the actin family of proteins (Clarke *et al.* 2007). Similarly, cluster 13 included genes associated with structural constituents of muscle (*P* = 0.00108), but in this case the smooth muscle that surrounds the GI tract. Genes in cluster 13 were predominantly expressed in the rumen complex of adult and 8 week old sheep, and included several genes related to smooth muscle function and regulation. *CALD1*, for example, encodes a calmodulin- and actin-binding protein that plays an essential role in the regulation of smooth muscle contraction (Huang *et al.* 2010). Intestinal motility is the result of contraction and relaxation of the smooth muscle, and a key function of the GI tract.

The numerous functions of the GI tract such as intestinal motility, exocrine and endocrine secretion, immune function and circulation are under complex neural control. Clusters 15 and 19 were associated with neuronal cells and included GO terms for synapse (*P* = 0.000018) and synaptic membrane (*P* = 4.9x10^−7^). However, overall expression of these genes was low relative to other clusters, presumably because neurons comprise only a small percentage of the cells that make up the GI tract, and therefore their expression level would be reduced compared to other more abundant cell types. The increased expression level at birth of genes in cluster 15 may be because comparatively fewer other cell types are differentiated at this time point.

### Strong immune signatures highlight the role of the GI tract as an immune organ

The intestinal epithelium, a single-cell layer, is the barrier between the external environment of the lumen (which contains pathogens, antigens and commensal bacteria) and the body. As such it is not surprising that the second largest cluster of genes (Figure 2 - Cluster 2) contained many genes associated with the immune response, their expression being two- to threefold higher in the ileum and Peyer’s patches (small masses of lymphatic tissue found throughout the ileum) than other regions of the GI tract. This pattern of expression was also observed in the pig GI tract (Freeman *et al.* 2012). The lower small intestine Peyer’s patches form an important part of the immune system by monitoring populations of intestinal bacteria and preventing the growth of pathogenic bacteria (Jung *et al.* 2010). Included in cluster 2 were genes encoding many of the protein components of the B- cell receptor complex (*CD19*, *CD79B*, *CR2*) (Treanor 2012). Also evident in this cluster were genes associated with the cell cycle, for example cyclins, DNA polymerases and kinesins, which were identified in the sheep gene expression atlas as a cell cycle specific cluster (Clark *et al.* 2017b). Significant GO terms for cluster 2 include G2/M transition of mitotic cell cycle (*P* = 1.8x10^−9^) and meiotic cell cycle process (*P* = 6.9x10^−9^). The high level of lymphocyte proliferation and replenishment of intestinal macrophages by a high turnover of monocytes (Bain & Mowat 2011; Shaw *et al.* 2018), and therefore the frequency of cells undergoing mitosis in the Peyer’s patches and ileum, might explain the association of cell cycle genes with an immune signature (David *et al.* 2003).

Other GI clusters with an immune signature included clusters 3, 7, 9 and 10. Cluster 3 exhibited a strong immune signature, particularly associated with T-cells, with significant GO terms for positive regulation of T-cell activation (*P* = 2.1x10^−21^) and cytokine receptor activity (*P* = 5.4x10^−5^). Genes within cluster 3 included the T-cell marker genes *CD3*, *CD4* and *CD6*, as well as Toll-like receptor genes *TLR1* and *TLR10* (Akira & Takeda 2004). Cluster 9 exhibited a similar immune related expression pattern including genes involved in T-cell-B-cell interactions such as *CD37*, and cytokine production such as *IL10* and *CD80*. Significant GO terms for cluster 9 included B- cell receptor signaling pathway (*P* = 4.9x10^−6^) and regulation of B-cell activation (*P* = 6.3x10^−6^) as well as Toll-like receptor 4 signaling pathway (*P* = 2.8x10^−5^). Macrophages play an essential role in inflammation and protective immunity and in the GI tract are important for local homeostasis, maintaining a balance between microbiota and the host immune system (Mowat and Bain 2011). Clusters 7 and 10 contained genes that are also commonly used as marker genes for intestinal macrophages including *CCRL1* (synonym *CX3CR1*), *ITGAX* (synonym *CD11C*) and *ITGAM* (synonym *CD11B*) (Mowat and Bain 2011; Bain and Mowat 2011; Shaw *et al.* 2018).

**Figure 3:**
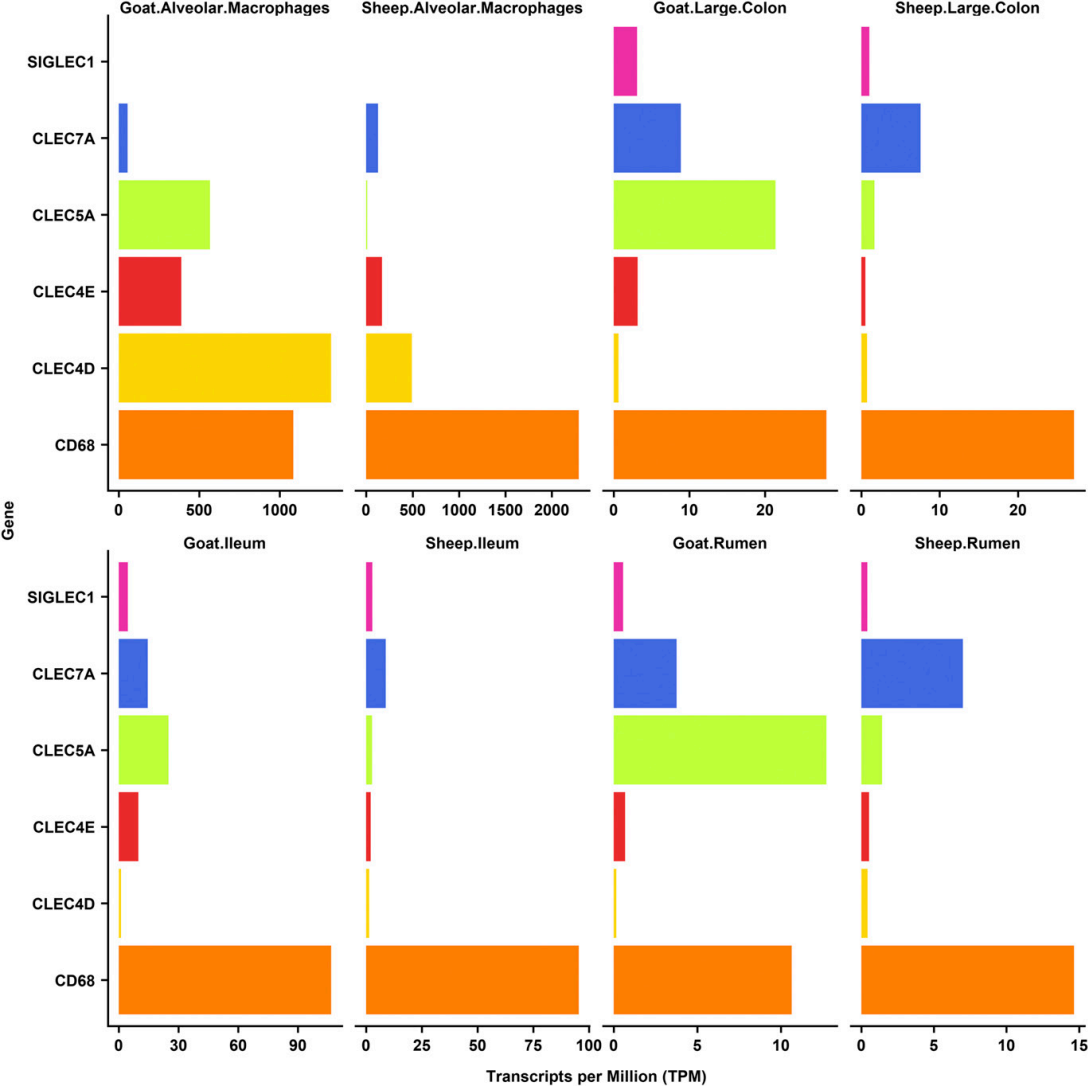
Comparative analysis of the expression of five C-type lectin genes, measured as transcripts per million (TPM), across three GI tract tissues (large colon, ileum and rumen) and alveolar macrophages in sheep and goats.

We included alveolar macrophages in the gene-to-gene network clustering of the GI tract dataset to look specifically at the regulated expression of receptors involved in bacterial recognition in the GI tract relative to their expression in other populations of macrophages. In the mouse, alveolar macrophages have a unique transcriptomic profile consistent with their function as the immediate host defense against inhaled pathogens, and their differentiation depends uniquely upon granulocyte-macrophage colony-stimulating factor (Guilliams *et al.* 2013). One such receptor, *SiglecF* (*Siglec5*), is expressed specifically in mouse alveolar macrophages and used as a marker for these cells. The same pattern receptors are down-regulated in the macrophages of the wall of the gut, which depend upon signals from the macrophage colony-stimulating factor (*CSF1*) and transforming growth factor beta (*TGFb*) (Mowat *et al.* 2017). Down-regulation of receptors involved in bacterial recognition is necessary to avoid chronic inflammation in response to lumenal microorganisms and food antigens. Consistent with this view, analysis of the pig gene expression atlas (Freeman *et al.* 2012) revealed that there were a large number of genes for C-type lectins that were highly-expressed in alveolar macrophages but almost undetectable in the pig GI tract despite the high expression of known macrophage-specific transcripts (Freeman *et al.* 2012). To determine if this was also the case in sheep we examined the expression of six C-type lectin marker genes (*CD68*, *CLEC4D*, *CLEC4E*, *CLEC5A*, *CLEC7A* and *SIGLEC1*) using the gene expression profiles for the sheep atlas dataset on BioGPS (Clark *et al.* 2017a). In sheep, as in pig, *CLEC4D*, *CD68*, *CLEC4E* and *CLEC7A* were all highly expressed in sheep alveolar macrophages, but barely detected in the GI tract tissues. *CLEC5A* and *SIGLEC1* did not show the same upregulation in sheep alveolar macrophages as they did in pigs indicating these genes exhibit a species-specific expression pattern. To investigate whether these trends also applied to other ruminants, we compared the expression of these genes in alveolar macrophages, ileum, large colon and rumen from one-week old sheep with age-matched goats (Figure 3). Similar expression patterns were observed for goat as for sheep, the only exception being *CLEC5A* which was highly expressed in goat alveolar macrophages.

The alveolar macrophage associated expression of *CD68*, *CLEC4D*, *CLEC4E* and *CLEC7A* suggests they are likely to be necessary for the elimination of inhaled pathogens. Consistent with this view, *CLEC4E* has been shown to be important in protective immunity against pneumococcal pneumonia in lung macrophages in mice (Behler-Janbeck *et al.* 2016) and *CLEC7A* is involved in the innate immune respiratory response to fungal pathogens (Saijo & Iwakura 2011). Although ruminants have a larger and more diverse microflora in the GI tract compared to pigs, which are monogastric mammals (O’ Donnell *et al.* 2017), expression patterns of the C-type lectin marker genes appear to be similar between the two species. Our findings generally support the conclusion that intestinal macrophages adapt to down-regulate their recognition of microorganisms (Freeman *et al.* 2012; Mowat *et al.* 2017).

### Network cluster analysis and principal component analysis of tissue samples reveals a strong effect of developmental stage on tissue specific transcription

**Figure 4:**
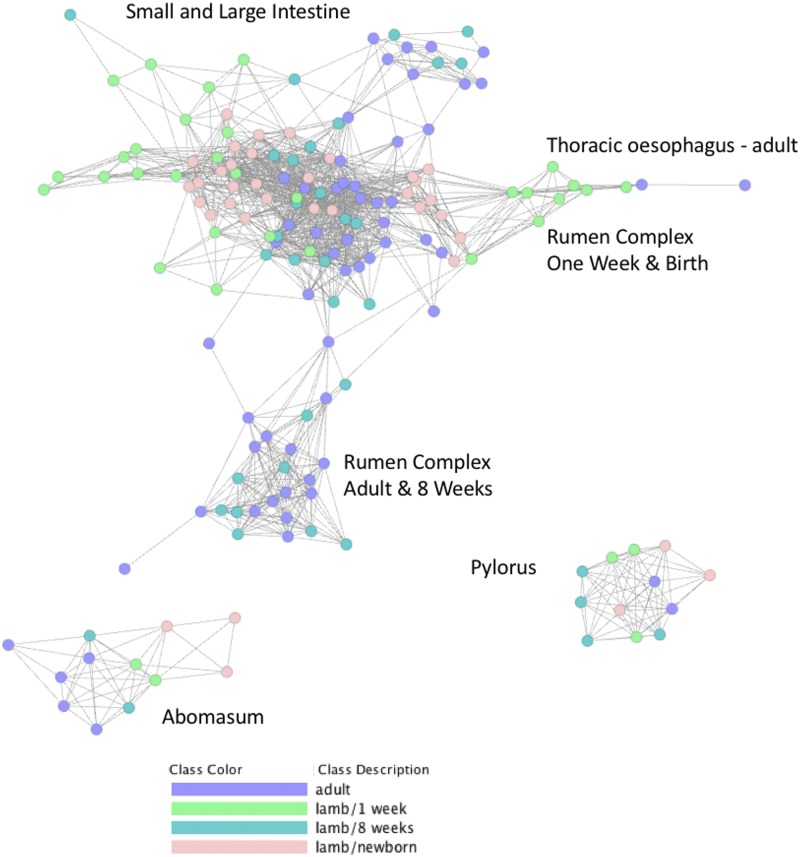
Sample-to-sample network graph of sheep GI tract samples colored according to developmental stage. Created using Graphia Professional with parameters Pearson’s R = 0.85, MCLi = 2.2, Minimum Component Size = 2, Minimum Cluster Size = 2.

Sample-to-sample network cluster analysis was used to visualize the effect of developmental stage on tissue specific gene expression. To perform the sample-to-sample network cluster analysis we used a version of the GI tract dataset that was not averaged across individuals and did not include the Texel dataset or the alveolar macrophage and liver samples (Figure 4). The full version of this dataset was published with the sheep gene expression atlas and is available for download through the University of Edinburgh DataShare portal (http://dx.doi.org/10.7488/ds/2112). A version only including the TPM estimates used for this analysis is included here as Table S4.

Developmental stage specific transcriptional patterns were reflected particularly strongly in the rumen complex (reticulum, rumen and omasum) tissue samples (Figure 4). Rumen complex samples from lambs at birth and one week of age formed a distinct cluster from comparable samples from 8 weeks of age and adult animals. This reflects the changes in tissue structure, function and morphology of the reticulum, rumen and omasum in the transition from pre-ruminant to ruminant. The close proximity of the adult esophageal samples and the pre-ruminant rumen complex samples in the network cluster analysis indicate that tissue from the rumen complex at birth and one week of age is transcriptionally similar to adult esophageal tissue in comparison with tissue from the rumen of adult sheep (Figure 4). At birth and one week of age the walls of the reticulum join together to form an ‘esophageal’ or ‘reticular’ groove transferring milk and colostrum directly to the abomasum, where it is digested efficiently bypassing the rumen (Figure 1) (Meale *et al.* 2017). Rumen tissue of lambs at birth and at one week of age will therefore be functionally and transcriptionally different from rumen tissue of adult sheep. The rumen of neonatal lambs, for example, is essentially non-functional in ketogenic capacity and does not exhibit the same high degree of keratinisation that is characteristic of the adult rumen (reviewed in (Baldwin *et al.* 2004)). Consequently, the strong epithelial and metabolic signatures of the adult rumen (Xiang *et al.* 2016b) are likely to be weaker at one week and birth driving some of the observed cluster separation between the developmental stages (Figure 4).

The sample-to-sample graph also illustrates the strong tissue specific differences in transcription (Figure 4). The pylorus and abomasum samples, for example, form distinct clusters from the other regions, reflecting the fact that they have unique functions. Both have a glandular lining, with various glandular cell types involved with enzyme secretion, lubrication, and endocrine control and specialized structures, such as the pyloric and fundic glands of the stomach (Dyce *et al.* 2010). Developmental stage- and tissue- specific transcriptional patterns observed in the sample-to-sample graph were also reflected in principal component analysis (PCA) of the dataset (Figure S1). Tissue specific clusters of samples were largely separated by organ system (rumen complex, small and large intestine, esophagus and ‘true’ stomach) (Figure S1 A&B). The effect of age on transcription was particularly evident in PC2, with samples from newborn lambs clustered separately from the other 3 time points (Figure S1 C&D).

### Developmental stage specific changes in immune gene signatures occur during the transition from pre-ruminant to ruminant

One of the largest macrophage populations in the body occupies the lamina propria of the GI tract (Bain & Mowat 2011). Macrophages are so numerous that the expression of macrophage-related genes can be detected within the total mRNA from GI tract samples. Constant exposure to food and environmental antigens and a wide diversity of commensal bacteria make the GI tract of developing sheep a primary site for pathogen infection (Mantovani & Marchesi 2014). As a consequence, GI immune cells are involved in a balanced immune response, focused on controlling pathogen invasion while recognizing commensal colonizing microbes. This response will change as the lambs age reflecting changes in diet (the transition from milk to pellets and hay) and environment (transition from maternal transmission of pathogens to those from the surrounding environment). Intestinal mononuclear phagocytes, of which monocyte-derived macrophages form the most abundant population play a key role in the maintenance of the intestinal immune response to pathogens and homeostasis (Mantovani & Marchesi 2014). Macrophage associated genes are therefore likely to be driving some of the observed transcriptional differences observed between developmental stages, described in the previous section (See Figure 4 and Figure S1).

**Figure 5:**
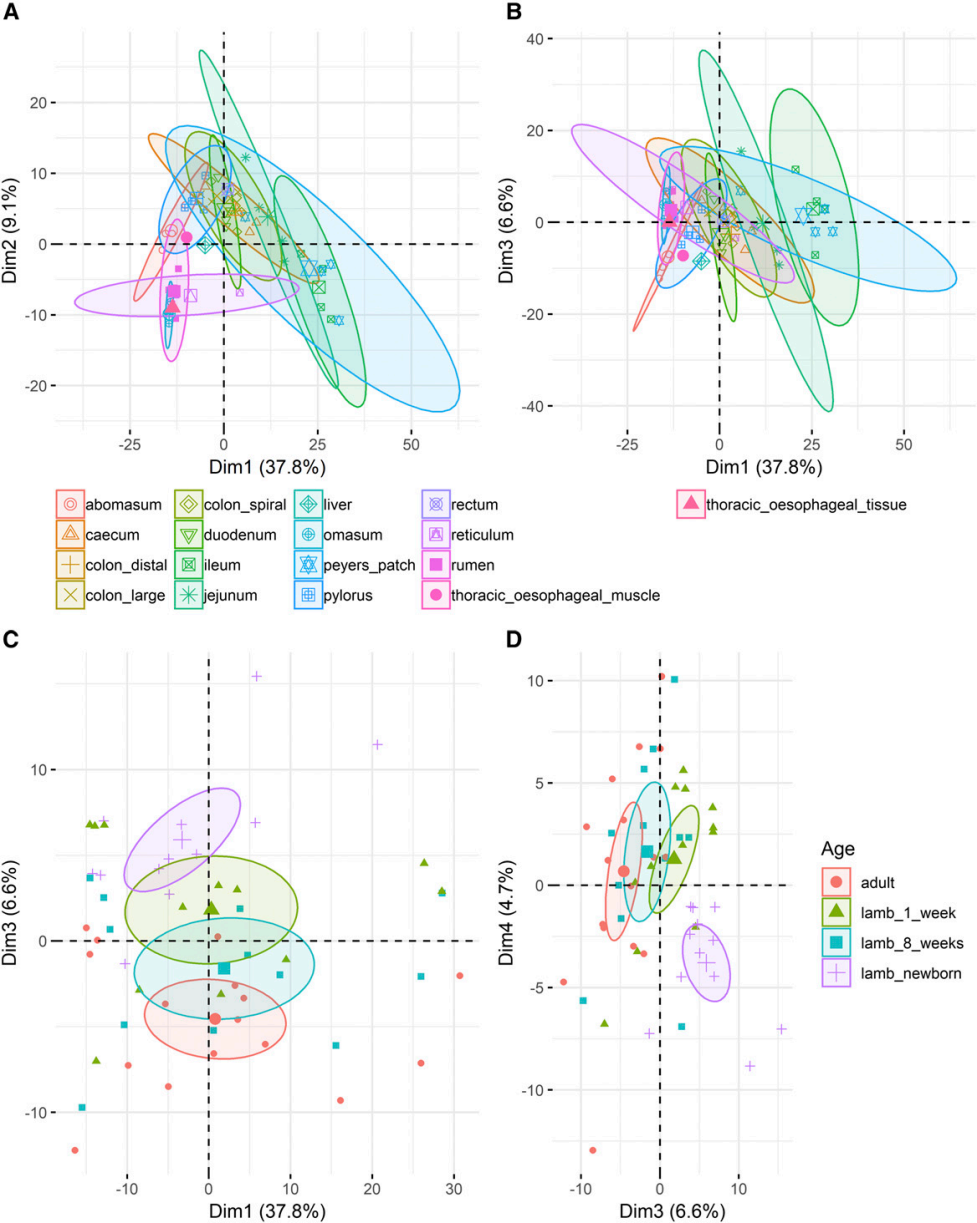
Principal Component Analysis of macrophage associated signatures in the sheep GI tract illustrating separation by tissue (A & B) and developmental stage (C & D) in three and four components, respectively. The mean of each group was used as the center of the circle colored-by-group with a confidence interval of 0.95 as the ellipse size.

We used PCA to examine the clustering pattern of a set of 490 macrophage- associated genes (extracted from the alveolar macrophage associated clusters 7 and 10 from the gene-to-gene network graph, detailed in Table S2) (Figure 5). We looked at tissue-specific transcriptional patterns (Figure 5A) and found that ileum, jejunum, duodenum and colon clustered separately from the rumen and reticulum in components PC1 and PC2, respectively, indicating differences in the macrophage expression profile in these tissues. A similar effect was observed between components PC1 and PC3 (Figure 5B). Developmental stage-specific transcriptional patterns in macrophage-associated genes were evident when comparing components PC1 and PC3 (Figure 5C), and particularly when comparing PC3 and PC4 (Figure 5D), where the samples from newborn lambs are clearly distinct from the other developmental stages. These differences might relate to the fact that newborn lambs would not have received any colostrum which provides the initial source of acquired immunity and is key in the homologous transfer of passive immunity between the mother and neonate (Stelwagen *et al.* 2009). A wide variety of immune components linked to the innate immune response have been identified in colostrum and milk including neutrophils and macrophages as well immunomodulatory factors (including numerous pro- and anti-inflammatory cytokines) and peptides and proteins with direct antimicrobial activity (Stelwagen *et al.* 2009). Another potential driver of these differences is that the GI tract will gradually become colonized with gut macrophages, and at birth there will be a comparatively higher proportion of monocytes (Shaw *et al.* 2018). The GI tract harbors multiple distinct populations of macrophages, monocytes and other immune cells that exhibit some level of stochasticity over time (Bain & Mowat 2011; Shaw *et al.* 2018) which is reflected in transcriptional patterns.

The expression of the monocyte marker gene *CD14* increased with age in the rumen, reticulum and omasum. This may reflect the lower number of monocytes in neonatal lambs relative to juvenile and adult animals (Kramer *et al.* 2003) or the high turnover of blood monocytes and continual replenishment of intestinal macrophages by monocytes that has been reported in the GI tract (Bain *et al.* 2016). *IL10* expression was very low across all the GI tract tissues in contrast to other mammalian species where it has been shown to be constitutively expressed in intestinal macrophages (reviewed in (Bain & Mowat 2011)). For the majority of tissues TPM was <3 with the exception of the ileum and Peyer’s patch where expression ranged between 6 and 12 TPM. In other species, interleukin 10 (*IL10*) is a key anti-inflammatory cytokine that can inhibit proinflammatory responses of both innate and adaptive immune cells (Mantovani & Marchesi 2014; Shouval *et al.* 2014). In mice, macrophage-specific conditional deletion of the *IL10* receptor causes severe spontaneous colitis (Zigmond *et al.* 2014) as does a full mutation of the *IL10* gene (Kühn *et al.* 1993). In the sheep atlas dataset on BioGPS, *IL10* mRNA is strongly induced in macrophages by lipopolysaccharide (Clark *et al.* 2017a). However, the induction is rapid and transient, and decays completely after 24 hr. We speculate that *IL10* is induced transiently in sheep monocytes that enter the intestinal lamina propria and are then repressed.

### Genes associated with metabolism, epithelia and connective tissue exhibit developmental stage-specific transcriptional signatures

To characterize which genes were driving transcriptional patterns, more broadly, during the transition from pre-ruminant to ruminant we chose three regions of the GI tract (rumen complex, stomach and small intestine) and selected one tissue per region (rumen, abomasum and ileum) to perform differential expression analysis using pairwise comparisons between birth and one week and one week and 8 weeks of age (Table S5 – Rumen, Table S6 – Ileum and Table S7 – Abomasum). To determine the main functions of genes exhibiting differential expression in each comparison we used GO term enrichment (Table S8). GO terms related to immunity were common across all three tissues but particularly the rumen and ileum between birth and one week of age. In the ileum between birth and one week of age enriched GO terms were predominantly associated with immunity and included ‘immune response’, ‘chemokine activity’, ‘chemokine production involved in the inflammatory response’ as well as others related to metabolism. In contrast between one and 8 weeks of age GO terms were associated predominantly with metabolic processes, and vesicle formation. A similar but less exaggerated trend was also observed in the rumen, with GO terms for ‘defense response to other organisms’, ‘immune response’ and more generally for metabolism enriched between birth and one week of age and then a shift toward metabolic and muscle and epithelial differentiation between one and 8 weeks. In the abomasum enriched GO terms between both sets of time points were more similar than for the other two tissues which might reflect the fact that the functional changes in the abomasum are comparatively less than the other two tissues.

The structural composition of the rumen and ileum changes significantly during the transition from pre-ruminant to ruminant, with the most dramatic physical changes associated with the rumen epithelium (Baldwin *et al.* 2004). Several genes associated with connective tissue and collagen were differentially expressed from 1 week to 8 weeks of age in the rumen. Genes exhibiting greater than twofold up-regulation included *COL1A1* and *COL1A2* (Table 2). Genes associated with keratinocytes and the epithelial signature of the rumen exhibited differential expression patterns according to developmental stage. *KRT36*, for example, showed a more than fourfold increase in expression between one and 8 weeks of age (Table 2). *KRT36* has been shown to exhibit significant transcriptional responses to changes in diet in dairy cattle (Li *et al.* 2015) and the change in diet between one and 8 weeks of age is likely to be driving at least a proportion of the observed expression patterns.

**Table 2:** Examples of differentially expressed genes identified using pairwise comparison of abomasum, rumen and ileum samples from sheep at one-week *vs.* newborn and 8 weeks *vs.* 1 week. The gene expression estimate (as TPM) at the earliest developmental stage in each comparison is included as TPM 1 and at the later developmental stage as TPM 2

Gene	Tissue	Developmental Stage	Fold Change	P-Value	Direction	TPM 1	TPM 2
***COL1A1***	Rumen	8 Weeks *vs.* 1 Week	2.1	3.3x10^−5^	Up	29,269	92,501
	Ileum	8 Weeks *vs.* 1 Week	−2.3	8.5x10^−15^	Down	35,506	6919
***COL1A2***	Rumen	8 Weeks *vs.* 1 Week	2.1	7.3x10^−5^	Up	25,919	80,206
	Ileum	8 Weeks *vs.* 1 Week	−2.1	5.6x10^−14^	Down	33,384	7541
***KRT36***	Rumen	8 Weeks *vs.* 1 Week	3.9	6.5x10^−8^	Up	6384	69,503
***HMGCS2***	Rumen	One Week *vs.* Newborn	8.4	2.3x10^−50^	Up	66	19,467
***SLC14A1***	Rumen	One Week *vs.* Newborn	2.4	9.3x10^−16^	Up	2181	10,158
***IL36A***	Rumen	8 Weeks *vs.* 1 Week	3.7	1.4x10^−5^	Up	275	2647
***CLEC4E***	Ileum	8 Weeks *vs.* 1 Week	2.4	2.7x10^−4^	Up	15	76
***PIP***	Rumen	8 Weeks *vs.* 1 Week	3.1	5.1x10^−7^	Up	375	2113
***IFIT2***	Ileum	One Week *vs.* Newborn	2.0	1.8x10^−6^	Up	76	275
***IFIT3***	Abomasum	8 Weeks *vs.* 1 Week	3.1	7.7x10^−31^	Up	337	439
***MX1***	Abomasum	8 Weeks *vs.* 1 Week	2.1	5.5x10^−21^	Up	1048	1307
	Rumen	One Week *vs.* Newborn	3.6	1.2x10^−5^	Up	220	2256
***DUOXA2***	Rumen	One Week *vs.* Newborn	4.1	3.2x10^−5^	Up	12	168
***IDO1***	Ileum	One Week *vs.* Newborn	4.2	2.0x10^−8^	Up	88	1467

Similarly, the mitochondrial enzyme encoding gene *HMGCS2*, which belongs to the HMG-Co synthase family and catalyses the first reaction in ketogenesis (Lane *et al.* 2002), was up-regulated eightfold in the rumen between birth and one week of age (Table 2). *HMGCS2* is differentially expressed in the calf rumen during the transition from pre-ruminant to ruminant (Connor *et al.* 2013; Kato *et al.* 2016) and in the rumen of developing lambs (Lane *et al.* 2002; Wang *et al.* 2016). It has been suggested that dietary changes through early development are likely to promote ketogenesis in rumen epithelial cells via PPAR-α-mediated activation of *HMGCS2* to promote papillary development, as well as activation of genes promoting fatty acid beta-oxidation to support cellular differentiation (Connor *et al.* 2013). *SLC14A1* has also been shown to be differentially expressed in the calf rumen during the transition from pre-ruminant to ruminant (Connor *et al.* 2013) and is differentially expressed in the rumen of lambs between birth and one week of age (Wang *et al.* 2016). In this study *SLC14A1* was upregulated over twofold between birth and 1 week of age in the rumen (Table 2). *SLC14A1* encodes a protein which mediates the basolateral cell membrane transport of urea, a key process in nitrogen secretion into the ruminant GI tract (Abdoun *et al.* 2010). A characteristic of ruminants is a high level of nitrogen recycling in the GI tract. Recycling of nitrogen via urea secretion into the rumen allows ruminants to survive on low-protein diets while at the same time producing milk and meat for human consumption (Abdoun *et al.* 2010). As such, *SLC14A1* and other genes involved in nitrogen recycling could be exploited to improve ruminant feed conversion efficiency, via nutritional intervention during development for example.

### Large differences in developmental stage specific expression patterns are associated with the immune response

Several genes involved in the immune response were differentially expressed in the rumen, ileum and abomasum of developing lambs during the transition from pre-ruminant to ruminant (Table 2). Many of these genes are likely to be part of the acute phase immune response, by regulating production of key cytokines such as *IL-6* and thus mediating activation of the NF-κB signaling pathways (Perkins 2007). *IL36A* shows almost a fourfold increase in expression between one and 8 weeks of age in the rumen and is thought to influence the skin inflammatory response by acting directly on keratinocytes and macrophages and indirectly on T-lymphocytes to drive tissue infiltration, cell maturation and cell proliferation (Foster *et al.* 2014). Other C-type lectins and genes involved in cytokine signaling also showed differential expression between the different developmental stages, which may be a consequence of increasing exposure to environmental factors. *CLEC4E*, for example, was more than twofold upregulated in the Ileum between one and 8 weeks of age.

Other immune genes, including *PIP* (Prolactin Induced Protein), were upregulated threefold in the rumen of lambs between one and 8 weeks of age. *PIP* has been shown to be preferentially expressed in the rumen of adult sheep (Xiang *et al.* 2016a), and is thought to play a role in mucosal immunity in ruminants (Hassan *et al.* 2008). Multiple IFN-inducible genes, including *IFIT2*, *IFIT3* and *MX1*, were differentially expressed in the abomasum, rumen and ileum during the transition from pre-ruminant to ruminant (Table 2). These genes - *IFIT2*, *IFIT3* and *MX1* - have recently been shown to be differentially expressed in sheep fibroblast cells in a type I IFN-induced antiviral state (Shaw *et al.* 2017). The differential expression patterns of these genes throughout the development of the rumen, abomasum and ileum highlights both their importance in the innate immune response and the role of the GI tract as an immunologically active site.

Several genes involved in both metabolism and immunity were differentially expressed during the transition from pre-ruminant to ruminant. *DUOXA2*, for example is involved in thyroid hormone synthesis and lactoperoxidase-mediated antimicrobial defense at the mucosal surface (Bae *et al.* 2010). The rumen is the main site of colonization by micro-organisms as the lamb develops. *DUOX2* and *DUOXA2*, which encode subunits of dual oxidase, have previously been shown to be upregulated in the rumen of adult sheep (Xiang *et al.* 2016a; Xiang *et al.* 2016b) and here we found *e*xpression of *DUOXA2* fourfold up-regulated in the rumen between birth and one week of age. *DUOXA2* might be involved in controlling microbial colonization as the lamb transitions from pre-ruminant to ruminant. Similarly, *IDO1* was more than fourfold upregulated in the ileum of lambs between birth and one week of age and has been implicated in immune modulation through its ability to limit T-cell function and engage mechanisms of immune tolerance (Grohmann *et al.* 2003; Plain *et al.* 2011). *IDO1* encodes indoleamine 2,3-dioxygenase (IDO) which is a heme enzyme that catalyzes the first and rate-limiting step in tryptophan catabolism to N-formyl-kynurenine (Grohmann *et al.* 2003). Through its expression in monocytes and macrophages this enzyme modulates T-cell behavior by its peri-cellular catabolization of the essential amino acid tryptophan (Munn & Mellor 2013). It has also been shown to be highly expressed in the jejunal mucosa of pre-weaning calves (Hammon *et al.* 2018). With further functional validation *DUOXA2* and *IDO1* might be suitable candidates for the development of novel therapeutics against microbial pathogens.

### Comparative analysis of the rumen, colon and ileum of one-week old age-matched sheep and goats reveals differences in expression of genes involved in metabolism and immunity

**Figure 6:**
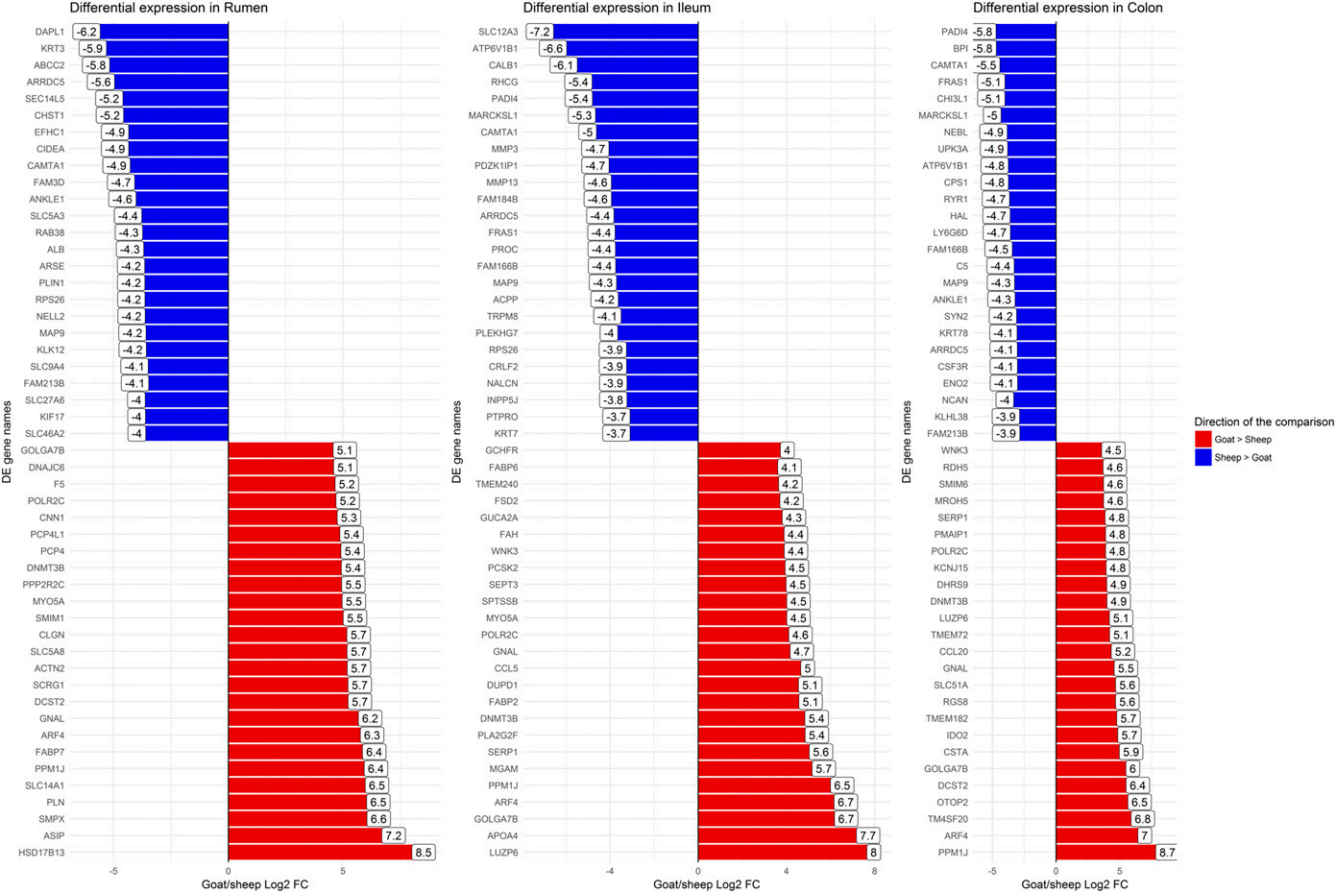
The top 25 differentially expressed genes up-regulated and top 25 down-regulated genes between goat and sheep in the rumen, ileum and colon of age-matched one-week old animals.

We performed a comparative analysis of the gene expression estimates for age-matched one-week old sheep and goats for three GI tract tissues: rumen, ileum and colon. The goat gene expression estimates as transcripts per million (TPM) both for these tissues are included in Table S9. Full lists of all differentially expressed genes between sheep and goat are included in Table S10 (rumen), Table S11 (ileum) and Table S12 (colon). The top 25 differentially expressed genes between sheep and goat in either direction for each of the three tissues are shown in Figure 6. Several immune genes were upregulated in the goat ileum including *CCL5*, one of the predominant cytokines expressed during damage and inflammation of epithelial keratinocytes (Wetzler *et al.* 2000). Other genes with metabolic and immune functions were differentially expressed, including *IDO2*, which was upregulated in the colon of one-week old goats relative to age-matched sheep. *IDO2*, with its paralogue *IDO1*, mentioned above, catalyses the first and rate-limiting step of the catabolism of the essential amino acid tryptophan along the kynurenine pathway (Grohmann *et al.* 2003) and is involved in the metabolic control of immune responses (Munn & Mellor 2013). IDO has been implicated in downregulating immune responses to *Mycobacterium avium* subsp. *paratuberculosis*, the causative agent of Johne’s disease (Plain *et al.* 2011). In sheep and cattle an increase in IDO expression correlates with progression to clinical mycobacterial disease (Plain *et al.* 2011) with the small intestine the primary site of infection (Koets *et al.* 2015). These differences in expression might underlie species-specific differences in disease susceptibility and response to pathogens. As such these genes could be potential targets for gene editing, initially in cell lines, and would be suitable candidates for further functional analysis.

Interestingly, *SLC14A1*, which was differentially expressed in the rumen of sheep between birth and one week of age, was also upregulated in the rumen of goats (Figure 6). As mentioned above the protein encoded by *SLC14A1* is involved in nitrogen secretion into the ruminant GI tract (Abdoun *et al.* 2010). Nitrogen recycling is an important topic in ruminant management because movement of urea across the GI tract can have major effects on metabolism (Lapierre & Lobley 2001) and nitrogenous content of urea between sheep and goats varies (Bristow *et al.* 1992). These genes would be candidates for further analysis to elucidate the functional significance of species-specific differences in gene expression. We do not know, however, if the rates at which sheep and goats mature vary and as such the expression profiles we have generated might actually reflect developmental stage rather than species specific differences. This caveat should be considered when interpreting these results.

### Conclusions

By characterizing tissue specific transcription in the GI tract through the transition from pre-ruminant to ruminant we have shown that there are significant developmental-stage specific differences in gene expression particularly between neonatal lambs and 8 week and adult sheep. These differences were most obvious in the rumen complex where significant morphological and physiological changes occur as the lamb transitions from a milk-based to a grass and pellet diet. Differences in the expression of protein coding genes with age were observed both when the whole transcriptome was included in the analysis, and also when only a subset of macrophage associated genes were analyzed.

The availability of high quality, highly contiguous, well annotated reference genomes for ruminant species, particularly for sheep (Jiang *et al.* 2014) and goat (Bickhart *et al.* 2017; Worley 2017) has helped significantly with our analysis. The sheep gene expression atlas dataset (Clark *et al.* 2017b) utilized for this study represents a major contribution to the international Functional Annotation of Animal Genomes Consortium (FAANG) (Andersson *et al.* 2015). We focused only on protein-coding genes in this study, and have presented a wider characterization of non-coding transcripts elsewhere (Bush *et al.* 2018). A low level, highly tissue-specific expression pattern is characteristic of lncRNAs in sheep and goats (Bush *et al.* 2018). Further characterization of the lncRNAs and other non-coding transcripts expressed specifically in the GI tract through development, might help to infer something about their function and would be an interesting direction for future work. Similarly, analysis of isoform regulation using RNA-Seq data, as in (Katz *et al.* 2010), in the GI tract tissues would also be useful.

The results of this study also provide a foundation for future studies linking gene expression with microbial colonization of the developing GI tract. Other studies have examined links between feeding regime, the host transcriptome and microbial diversity in sheep using sequencing analysis of 16S rRNA genes (Wang *et al.* 2016). A full characterization linking tissue- and developmental stage-specific microbial colonization of the GI tract would require a detailed 16S sequencing metagenomic approach as in (Wallace *et al.* 2015). Unfortunately, we did not collect samples for this study to address changes in microbial communities over time. Similarly, we also did not collect data for digesta, ruminal fluid, rumen composition or feed intake, which could also be correlated with the observed expression patterns. The design of future studies should build on the foundation we have provided by analyzing transcriptional profiles in parallel with the above parameters to provide a multidimensional picture of the drivers of gene expression during development.

The results we present in this study lay a foundation for further work, providing baseline estimates of gene expression in the GI tract at the whole tissue level from healthy lambs. As indicated by Connor *et al.* (2010) the use of techniques such as laser-capture microdissection will be needed to further characterize expression profiles of individual cell types within the GI tract, and to remove expression biases that may occur in studies evaluating whole tissue samples. Cutting edge single-cell RNA-Seq technology provides the solution to this, allowing a cell-specific level of resolution of gene expression profiles. Single-cell messenger RNA-Seq has already been applied to cells from mouse GI tract organoids revealing rare cell types (Grün *et al.* 2015) and the technology will hopefully be applied in the future to cells from the GI tract of ruminants, since *in vitro* systems are now available (Hamilton *et al.* 2018).

The development and establishment of the rumen has a major effect on metabolism, immunity and physiological processes in other GI tract tissues (Baldwin *et al.* 2004), which is reflected in the extensive transcriptional complexity observed in this study. Using this sub-set of RNA-Seq data, from our high resolution of atlas of gene expression in sheep (Clark *et al.* 2017b) we have improved our understanding of gene expression during the transition between pre-ruminant and ruminant and identified key genes that could be exploited to improve productivity in sheep and other ruminants as novel therapeutics or as potential targets for gene editing.
